# Assessment of the pterygoid hamulus dimensions and morphology in Iranian women using cone-beam computed tomography

**DOI:** 10.34172/joddd.40976

**Published:** 2024-12-14

**Authors:** Zahra Mahdavi, Ladan Hafezi

**Affiliations:** ^1^School of Dentistry, Islamic Azad University of Medical Sciences, Tehran, Iran; ^2^Department of Maxillofacial Radiology, Faculty of Dentistry, Islamic Azad University of Medical Sciences, Tehran, Iran

**Keywords:** Age groups, Cone-beam computed tomography, Morphology, Pterygoid hamulus

## Abstract

**Background.:**

Understanding the characteristics of the pterygoid hamulus (PH) is crucial for diagnosing and treating various oropharyngeal and craniofacial conditions. It also aids in interpreting radiographs and diagnosing unexplained oropharyngeal pains. Cone-beam computed tomography (CBCT) is a valuable tool, offering clinical insights into this previously understudied area. Accordingly, this study aimed to evaluate the morphology and dimensions of the PH and its changes by ageing in 20-40-year-old women in the Dental Branch of Islamic Azad University of Sciences, Tehran, Iran.

**Methods.:**

This cross-sectional study was conducted on CBCT scans from 258 women aged 20-40. The morphology, length, width, and angle of the PH in coronal and sagittal planes were investigated.

**Results.:**

The average length of the right and left hamulus was 5.50±1.37 and 5.37±1.36 mm. The average width of the right and left hamulus was 2.16±0.72 and 2.11±1.06 mm. The average coronal angle of the right and left hamulus was 22.3±9.79° and 30.16±8.99°. The average sagittal angle of the right and left hamulus was 26.11±7.26° and 26.34±7.63°. In contrast with the sagittal angle, the right and left hamulus’s length, width, and coronal angles were not symmetrical. Slender morphology was the most frequent morphology, and it was symmetrical only in the 20-30-year-old age group. Finally, no variable was affected by ageing.

**Conclusion.:**

Hamulus dimensions and morphology did not change with ageing in women aged 20-40. The PH symmetry varied by age group. Also, CBCT was a suitable tool for investigating hamulus changes.

## Introduction

 The pterygoid hamulus (PH) is a curved, small, and hook-like process with a slightly outward and forward direction extending from the inferior end of the medial pterygoid plate of the sphenoid bone.^1,2^ This process has a unique shape and is a crucial part of the skull base, yet it still needs to be more adequately represented on anatomical charts. Due to its proximity to the maxilla and oropharynx regions, PH has attracted the interest of many different fields.^1,3^ The normal function of several muscles, including tensor veli palatini (TVP), palatopharyngeus, and upper part of the superior pharyngeal constrictor (pars pterygopharyngea), depends on the position, size, and inclination of PH.^4^ Throughout a person’s growth and development, these muscles are primarily responsible for maintaining the separation between the nasal and oral cavities during swallowing and sucking.^3,5^ An elongated PH is associated with pterygoid hamulus syndrome (PHS), which is characterised by various signs and symptoms in the soft palate and pharynx regions and causes pain and discomfort, especially during swallowing.^3^ In 1987, Hjørting-Hansen and Lous introduced the term “pterygoid hamulus syndrome” to refer to discomfort in the palatal and pharyngeal areas resulting from an irregularly shaped PH.^6,7^ If a patient feels pain in these areas, it may be necessary to consider PHS as a potential differential diagnosis. On the other hand, insufficient growth of PH in newborns causes inadequate support of the cephalopharyngeal muscle and uncontrolled narrowing of the superior pharynx, resulting in problems like sleep apnoea and snoring.^3^

 The knowledge of PH morphology and characteristics is essential and helpful for interpreting head and neck radiographs, including cephalometric radiography, submentovertex view, and Waters view. It provides valuable information for the differential diagnosis of oral and pharynx pains, particularly those without a known aetiology. One of the pioneers in evaluating PH morphology was the study by Eyrich et al in 1997.^8^ Identification of this process using conventional radiography is possible but remarkably difficult due to superimpositions and distortions. In contrast, cone-beam computed tomography (CBCT) offers a more precise visualisation of craniofacial structures through 3-dimensional (3D) views from various planes.^4^

 Despite the importance of understanding PH morphology, few studies have evaluated the morphological features and dimensions of PH, particularly in Iranian society. Therefore, the current study evaluated PH morphology and dimensions using CBCT among 20-40-year-old female patients treated at the Dental Branch of Islamic Azad University of Sciences, Tehran, Iran.

## Methods

###  Study design

 An analytical cross-sectional observational study.

###  Setting and participants

 The CBCT scans of female patients between the ages of 20-40 years old who were referred to the radiology department of the Islamic Azad University of Medical Sciences from 9^th^ December, 2021 until 21^st^ September, 2022 were evaluated. All scans in which the posterior region of the maxilla was covered were included. CBCT images with unclear borders of the PH, blurred or low-quality images, images with artefacts that cover the region of interest, and images taken from patients who have had maxillofacial trauma such as PH fracture, traumatic maxillary third molar extraction, pathologic conditions in the posterior maxillary region, and bone deficiencies, including osteoporosis and familial or traumatic skeletal asymmetries, were excluded from the study.

 The scans were acquired using the Villa Sistemi Medicali device (Rotograph EVO 3D, Rome, Italy) while the patients were in standard position with an image acquisition time of 15 seconds. The exposure parameters were set at 230 kVp, 7 mA, and the required field of view had a voxel size of 8.5 μm × 8.5 μm × 8.5 μm. The images were exported in the Digital Imaging and Communications in Medicine (DICOM) format.

###  Study size

 Following the aforementioned eligibility criteria, the CBCT scans from 258 patients were included in the study.

###  Variables

**Figure 1:**
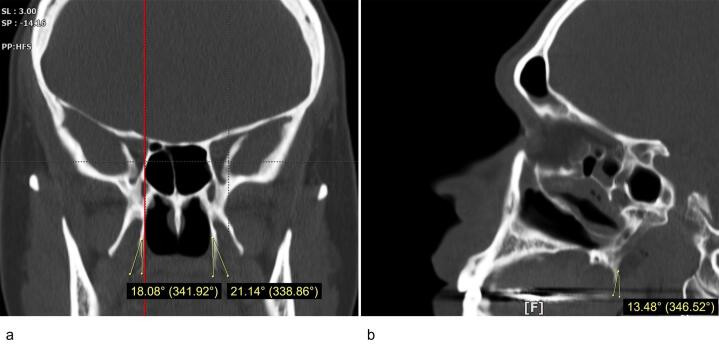
The inclination of the pterygoid hamulus: (a) coronal plane, (b) sagittal plane

 Measurements of the size (length and width) and the inclination of the PH on the coronal and sagittal planes were conducted using the OnDemand 3D Viewer software (available at https://www.ondemand3d.com). The coronal sections were used to measure the length and width of the PH. In order to identify the junctional point between the PH and the pterygoid bone, the Multi-Planar Reformat tool was used to compare the coronal and sagittal views of the same side. The PH length was defined as the longest line connecting the medial pterygoid-PH junction to the tip of the PH. The distance between the most prominent points on the lateral and medial sides of the process was measured as the PH width.^9^ The coronal inclination of the PH was defined as the mediolateral angle between the PH long axis and the long axis of the medial pterygoid plane. The PH sagittal inclination was defined as the posteroanterior angle between the long axis of the PH and the long axis of the Medial pterygoid plane (Figure 1). The PH morphology was categorized as “Slender” or “Triangle” type according to the study by Nerkar et al.^10^ Examiner percentage error was calculated by repeating 5% of the measurements after two weeks.

###  Statistical methods

 Statistical analyses were performed using the Statistical Package for Social Sciences (SPSS) application for Windows, Version 25.0 (SPSS Inc., Chicago, IL, USA). The homogeneity of the variances was evaluated using the Kolmogorov-Smirnov test. An independent t-test was used to compare numeric variances with normal distribution between the age groups; otherwise, the comparison was made using the Mann-Whitney U test. Moreover, the Wilcoxon signed-rank test was used to conduct the symmetrical analysis of PH size and coronal and sagittal inclinations. The chi-square and McNemar’s analyses were used to compare the morphology between the age groups and the symmetry evaluations. Finally, *P* value < 0.05 was considered statistically significant.

## Results

###  Basic characteristics

 A total of 258 CBCT scans from female patients with a mean age of 32.90 ± 5.47 years were assessed in this study. Seventy-nine (31.23%) patients were aged between 21-30, and 174 (68.77%) were between 31-40 years old.

###  Size and inclination

 The coronal sections were used to measure the length and width of the PH on the right and left sides, while its inclination was measured on both coronal and sagittal views. The mean measured values of the PH size and inclination are presented in Table 1. According to the independent t-test and Mann-Whitney U analyses presented in Table 2, there was no significant difference between the two age groups regarding the PH size (length and width) and coronal and sagittal inclinations (*P* > 0.05).

**Table 1:** The mean measured values of the pterygoid hamulus size and inclination

**Variable**		**Minimum**	**Maximum**	**Mean±SD**
Left side	Length (mm)	2.27	9.39	5.37 ± 1.36
	Width (mm)	0.76	15.04	2.11 ± 1.06
	Coronal inclination (°)	4.40	58.70	30.16 ± 8.99
	Sagittal inclination (°)	9.70	49.70	26.34 ± 7.63
Right side	Length (mm)	1.69	10.80	5.50 ± 1.37
	Width (mm)	0.83	6.16	2.16 ± 0.72
	Coronal inclination (°)	8.20	66.00	32.30 ± 9.79
	Sagittal inclination (°)	6.60	52.60	26.11 ± 7.26

**Table 2:** Comparison of the pterygoid hamulus size and inclination on the right and left sides between the two age groups

**Variable**		**Age group (y)**	**Mean**	***P*** ** value**
Left Side	Length (mm)	20-30	5.15 ± 1.03	0.838
		31-40	5.47 ± 1.32	
	Width (mm)	20-30	1.72 ± 0.44	0.699
		31-40	2.29 ± 1.99	
	Coronal inclination (°)	20-30	29.18 ± 9.24	0.244
		31-40	30.60 ± 8.86	
	Sagittal inclination (°)	20-30	26.31 ± 7.66	0.828
		31-40	26.35 ± 7.64	
Right Side	Length (mm)	20-30	5.46 ± 1.57	0.506
		31-40	5.89 ± 3.76	
	Width (mm)	20-30	2.06 ± 0.67	0.089
		31-40	2.56 ± 3.36	
	Coronal inclination (°)	20-30	31.40 ± 9.31	0.323
		31-40	32.71 ± 10.00	
	Sagittal Inclination (°)	20-30	26.20 ± 7.04	0.927
		31-40	26.06 ± 7.37	

 There was a statistically significant difference in the length (*P* = 0.045), width (*P* = 0.008), and coronal inclination (*P* < 0.001) of the PH between the right and left sides, indicating that the PH is asymmetrical regarding these variables. In contrast, the Wilcoxon signed-rank test did not show a significant difference in the sagittal inclination of right and left side PHs (*P* = 0.427), so the sagittal inclination of PH was symmetrical. The PHs of the first age group (20-30 years old) had a symmetrical length (*P* = 0.556), width (*P* = 0.558), and sagittal inclination (*P* = 0.857), while the second age group (31-40 years old) were only symmetrical in terms of the sagittal inclination (*P* = 0.391)

###  Morphology

 The slender type PH was the most frequent morphology on both sides (left: 61.26%, right: 57.31%). The chi-square analysis did not show a significant difference in the left (P = 0.697) and right-side (*P* = 0.940) PH morphologies when the two age groups were compared (Table 3). Contrary to the first age group (20-30 years old) (*P* = 0.302), patients in the second age group (31-40 years old) had asymmetrical PH morphology (*P* = 0.002) (Table 4).

**Table 3:** Comparison of the pterygoid hamulus morphologies on the right and left sides between the two age groups

**Morphology**^a^		**Age group (y)**		***P*** ** value**
		**20-30 **	**31-40 **	
Left side	Slender type	47 (59.50)	108 (62.10)	0.697
	Triangle type	32 (40.50)	66 (37.90)	
Right side	Slender type	45 (57.00)	100 (57.50)	0.940
	Triangle type	34 (43.00)	74 (42.50)	

^a^ Reported as frequency (percentage).

**Table 4:** Comparison of the pterygoid hamulus morphological symmetry

**Age group (y)**	**Left side**	**Right side**		***P*** ** value**
		**Slender type**^a^	**Triangle type**^a^	
20-30	Slender type^a^	29	18	0.302
	Triangle type^a^	16	16	
31-40	Slender type^a^	72	36	0.002
	Triangle type^a^	38	28	

^a^ Reported as frequency.

## Discussion

 The correlation between the hamulus process and the posterior throat and oropharyngeal region pains was first evaluated in 1989 by Salins andBloxham^11^ and in 1996 by Shankland.^12^ Position, inclination and dimensions of the hamulus process significantly impact the normal function of the surrounding muscles. When these muscles are contracted, the oral cavity separates from the nasal cavity, and swallowing occurs.^4^ Since the tubar part of the TVP muscle is linked to the PH, the position and morphology of this process directly affect the function of the TVP muscle and, consequently, the function of the hard palate during swallowing. Furthermore, the PH inclination plays a vital role in a better tension of palatal aponeurosis.^3^

 The morphological alterations of the PH, such as growth deficiency in newborns, result in insufficient muscle support, followed by the uncontrolled narrowing of the upper pharynx, sleep apnoea and snoring.^13^ In addition, these changes might be misdiagnosed as temporomandibular disorders or glossopharyngeal neuralgia by mimicking symptoms such as ear pain, pain during chewing and swallowing, and oedema and erythema in the palate’s posterior region.^3,14^ PHS, known as the palate and pharynx pains caused by inflammation in the hamular region, is primarily due to hamulus process elongation. These pains might be misjudged with those of impacted third molar or middle ear cysts or tumours.^15^ Also, there is a risk of iatrogenic trauma to the PH during certain maxillofacial surgeries, especially posterior alveolar process operations (e.g., maxillary impacted third molar extraction).^16^

 Dentists must be well-versed in PH anatomical features and functions to diagnose, treat, and prevent maxillofacial pains and iatrogenic surgical traumas properly. They should consider PH-related disorders as a potential differential diagnosis, especially for pains with unknown aetiology. Hence, PH was studied for its morphometric characteristics and its alterations with age in the current study. Unlike the previous studies, we evaluated the effect of all variables simultaneously by measuring the size and inclination of PH on coronal and sagittal sections of 258 CBCT scans on both the right and left sides. Additionally, the PH symmetry was analysed in each age group. Consequently, our findings can help diagnose untraceable pains in the nasal and oropharyngeal regions. Notably, we tried to increase the results’ accuracy by limiting the samples to 20-40-year-old women.

 The measurements showed an average length of 5.50 ± 1.37 mm and 5.37 ± 1.36 mm for right and left PH and an average width of 2.16 ± 0.72 mm and 2.11 ± 1.06 mm, respectively. The coronal and sagittal inclinations of right and left PH were 22.3 ± 9.79°, 30.16 ± 8.99°, and 26.11 ± 7.26°, 26.21 ± 7.80°. There was no significant difference in PH length, width, and coronal and sagittal inclinations between the two age groups, indicating that the PH morphometric parameters did not alter with ageing. Our results were similar to those of the studies by Eyrich et al^8^ and Orhan et al,^4^ who reported that the right and left PH lengths were 4.5 and 5 mm, and 5.40 ± 2.00 and 5.48 ± 1.94 mm, respectively. Orhan et al also measured the PH width on the sagittal plane and reported an average width of 1.87 ± 1.17 and 1.72 ± 0.94 mm for right and left PHs.^4^ In contrast, in this study, the PH width was measured on coronal sections.

 Our study used the pterygoid plane’s long axis instead of the true horizontal line^3,4,17^ to assess the PH’s inclinations. However, the lateral and posterior inclinations of PH on coronal and sagittal sections were similar to previous investigations.^3,4,16^ In accordance with our findings, Komarnitki et al found a weak correlation between age and PH dimensional changes.^16^ Mehra et al evaluated the PH size in three age groups: < 20 years old, 20-50 years old, and > 50 years old. They reported increased PH length with age, while widths initially decreased, then increased.^1^ In addition, Romoozi et al stated that rather than the coronal inclination, the length and width of the PH on the sagittal plane significantly depend on age, so the PH length in 21-59-year-old adults increased compared to 15-21-year-old individuals.^13^ On the other hand, patients aged between 60 and 100 years old had shorter PH than younger adults. They also reported a reduction in PH width and sagittal inclination with age. A significant increase in PH length among adults (21-59 years old) compared to children (0-9 years old) and a subsequent reduction in the elderly (60-100 years old) by an average of 0.5 mm was also reported by Krmpotić-Nemanić et al.^3^ It has been noted by Orhan et al that although the association between PH size and age is not significant, older people generally have smaller PH than younger people. In line with our findings, PH did not change in size and inclination during the age of 20-40 years in all previous investigations.^4^

 Based on our symmetry analysis, the right and left sides’ PHs were only symmetrical in terms of sagittal inclination. According to Orhan et al, the left side PH was wider than the right in both males and females. However, there was no difference in the lengths of the right and left PHs.^4^ There are also other studies that have found the PH’s length and width symmetrical.^16^ The fewer participants and the absence of gender exclusivity could explain the difference in the results. Currently, there is no explicit explanation for the asymmetry of PH widths. Nevertheless, several factors may contribute to the observed asymmetry in length and width, including genetic factors and functional differences in oral habits, such as masticatory patterns and occlusal forces between the right and left sides. The variation in coronal inclination may result from asymmetrical growth patterns and development during embryogenesis. The recognition of such asymmetry in the PH is of clinical importance in various dental and maxillofacial procedures, including orthodontic treatments, prosthodontics, and surgical interventions. Dentists and oral surgeons should consider these anatomical variations when planning treatment modalities or surgical procedures that involve the PH.

 The findings of the current study revealed that the most common PH morphology on both sides was the slender type. However, the differences in the two morphologies’ frequencies were not significant when age groups were compared. In addition, a comparison of the right and left sides showed that the PHs were symmetrical regarding morphology. In contrast to the first age group (20-30 years old), patients between the ages of 31 and 40 showed asymmetrical PH morphology. Previously, Nerkar et al have reported that triangular morphology was the most prevalent form in Indian society.^10^ This difference can be explained by racial variations.

 It is essential to acknowledge the limitations of this study, such as its focus on a specific population (Iranian women aged 20-40). There could have been errors in the comparison results due to the uneven distribution of participants between age groups. In order to strengthen the generalisability of the results in future studies, we suggest evaluating a larger population with data gathered from multiple radiographic centres rather than from a single centre. Future research could explore potential factors influencing PH asymmetry, such as genetics, environmental factors, and functional habits.

## Conclusion

 Hamulus process morphometric characteristics did not change with age in women aged 20-40. On the other hand, age-related differences in PH symmetry were observed between the two groups. Finally, CBCT was an appropriate method for studying hamulus alterations.

## Competing Interests

 The authors declare there is no conflict of interests, whatsoever.

## Data Availability Statement

 All data produced in the present study are available upon reasonable request to the authors.

## Ethical Approval

 This study was carried out according to the ethical principles of the Declaration of Helsinki, in addition to local ethical and legal requirements. Ethics approval was obtained from the institutional research ethics committee of the Islamic Azad University of Medical Sciences (ID: IR.IAU.TMU.REC.1400.230). The need for written consent was waived by the Institutional Review Board of Islamic Azad University of Medical Sciences based on national regulations and the nature of this study. This study strictly adheres to de-identification techniques, ensuring the anonymity and confidentiality of all participants. Notably, it involves observational methods, and the study techniques employed are entirely harmless and burden-less to participants. Also, before participating in this research, individuals verbally consented to their de-identified data being used strictly for research purposes.

## Patients and Public Partnership

 The patients or the public were not involved in our research’s design, conduct, reporting, or dissemination plans.

## Transparency Declaration

 Authors declare that the manuscript is honest, accurate, and transparent. No important aspect of the study is omitted.
